# Association Between Four Anthropometric Indexes and Metabolic Syndrome in US Adults

**DOI:** 10.3389/fendo.2022.889785

**Published:** 2022-05-24

**Authors:** Yaling Li, Rui Zheng, Shuting Li, Ruyi Cai, Feihua Ni, Huiyan Zheng, Ruying Hu, Ting Sun

**Affiliations:** ^1^ Department Health Management Center, the Second Affiliated Hospital, School of Medicine, Zhejiang University, Hangzhou, China; ^2^ Department of Critical Care Medicine, Sir Run Run Shaw Hospital, School of Medicine, Zhejiang University, Hangzhou, China

**Keywords:** metabolic syndrome, triglyceride and glucose index (TyG), adiposity index (VAI), lipid accumulation product (LAP), waist-triglyceride index (WTI), anthropometric indexes

## Abstract

**Objective:**

To study the association between anthropometric indexes [lipid accumulation products (LAP), visceral obesity index (VAI), triglyceride and glucose index (TyG) and waist triglyceride index (WTI)] and metabolic syndrome (MetS) in a representative sample of American adult population surveyed by National Health and Nutrition Examination Survey (NHANES).

**Methods:**

Cross-sectional data from the NHANES were used. Participants were adults aged 18–80 y from 1996–2006. MetS were defined by the updated National Cholesterol Education Program/Adult Treatment Panel III criteria (NCEP-ATP III) for Americans. Receiver operating characteristic (ROC) curve was drawn and the areas under the curve (AUC) were used to assess the ability of these indexes in screening MetS. Statistical differences among the AUC values of these indexes were compared. The association between the anthropometric indexes and MetS was investigated using weighted multivariable-adjusted logistic regression.

**Results:**

560 (35.2%) males and 529 (26.4%) females were diagnosed with MetS. LAP was the strongest predictor of MetS for men (AUC=0.87, 95% CI 0.85-0.89), and also was the strongest for women [AUC=0.85, 95% confidence interval (CI) 0.83-0.86], according to the ROC curve analysis. In men, differences in AUC values between LAP and other anthropometric indicators were also significant (all *P*<0.001). In women, there was a significant difference in AUC values between LAP and WTI (*P*<0.001), but differences in AUC values between LAP and TyG, VAI were not significant.

**Conclusion:**

The present study indicated that LAP is a better predictor in the clinical setting for identifying individuals with MetS in the American adult population.

## Introduction

Metabolic syndrome (MetS) is a complicated disorder characterized by impaired glucose tolerance, dyslipidemia, elevated blood pressure, abdominal obesity (1–3). MetS is associated with higher risks of cardiovascular diseases, type 2 diabetes, some cancers, and all-cause mortality and has become one of the major challenges facing global and national public health institutions (4, 5). According to the National Health and Nutrition Examination Survey, more than one-third of adults suffer from MetS (6).

Obesity as the core manifestation of MetS has attracted more and more attention (7). There is some evidence to support the assumption that abdominal visceral fat has a stronger correlation with MetS (8, 9). Hence, it is reasonable to define visceral fat as a predictor of MetS. Magnetic resonance imaging (MRI) and computed tomography (CT) are considered as the gold standard for evaluating visceral fat (10). However, they cannot be used in epidemiological studies and clinical routine due to expensive, time-consuming, and exposure patients to radiation and contrast agents. Thus, it is very important to identify a simple and clinically suitable visceral obesity substitute indicator. Body mass index (BMI) is the most commonly used indicator of obesity, but it has limitations in assessing fat distribution (11, 12). Therefore, anthropometric indexes have been suggested to evaluate the amount and location of body fat to track metabolic disorders (13). Recently, visceral obesity index (VAI) and lipid accumulation products (LAP) have been recommended as reliable indicators of visceral obesity. VAI is calculated based on waist circumference (WC), high-density lipoprotein cholesterol (HDL-C), BMI, and triglyceride (TG) and has a separate formula for men and women (14). It has been reported to have a good ability to predict metabolic syndrome in Chinese and Iranian populations (15, 16). LAP is an index of abdominal fat over-accumulation based on TG and WC (13), which is considered as the best predictor of MetS in middle-aged and elderly people in Korea (17).

Insulin resistance (IR) is another core issue of MetS (7). Hyperinsulinaemic-euglycaemic clamp (HEC) is the gold standard for testing IR (18), but this approach is time-consuming and is not suitable for clinical application. The triglyceride and glucose (TyG) index combined with fasting plasma glucose (FPG) and TG has been proposed as an effective substitute for IR and has been reported to have a good predictive ability for MetS in Korean and Chinese populations (17, 19). Recently, inspired by the formula of the TyG index, Liu et al. combined WC with TG to develop a new index called waist-triglyceride index (WTI), which showed a strong ability to distinguish MetS (20).

Even though several papers on the association between anthropometric indicators and the MetS have been published (13), it is still hard to determine explicitly which indicator is the most predictable indicator of MetS. And these studies have limitations in adjusting confounding factors, most of them do not adjust the factors that may affect MetS, such as smoking, drinking, exercise, and socioeconomic factors (21). Postmenopausal women tend to deposit more visceral fat have shown by a large number of studies. Thus, gender may affect the relationship between anthropometric indicators and MetS. As far as we know, up to now, there are limited research on comparing anthropometric indicators of the American population with the predicted strength of MetS by gender. Accordingly, the purpose of this study was to investigate the relationship between anthropometric indicators (VAI, LAP, TyG, WTI) and MetS in American adults and to compare their predictive ability according to gender.

## Materials and Methods

### Data Source

The NHANES is a repeated national representative cross-sectional health examination survey conducted in the United States (US), on behalf of the non-institutionalized population of the US civilian population, which provides estimate of the lifestyle, nutritional status, and health of the US civilian population (22). Since 1999, NHANES has become a continuous survey, with data released every two years. During the survey, participants will complete a questionnaire survey, a series of tests, and offer blood and other biological samples at the mobile screening center (23). Five main parts make up the NHANES database, including demographic, questionnaire, laboratory, diet, and examination data.

More details are available on the official website (https://www.cdc.g-ov/nchs/nhanes/index.htm). The NHANES datasets (1999–2006) were downloaded from DataDryad (https://doi.org/10.5061/dryad.d5h62). Participants provided written informed consents. The National Center for Health Statistics (NCHS) Ethics Review Board approved the collection of the NHANES data.

### Participants Selection

**Figure 1:**
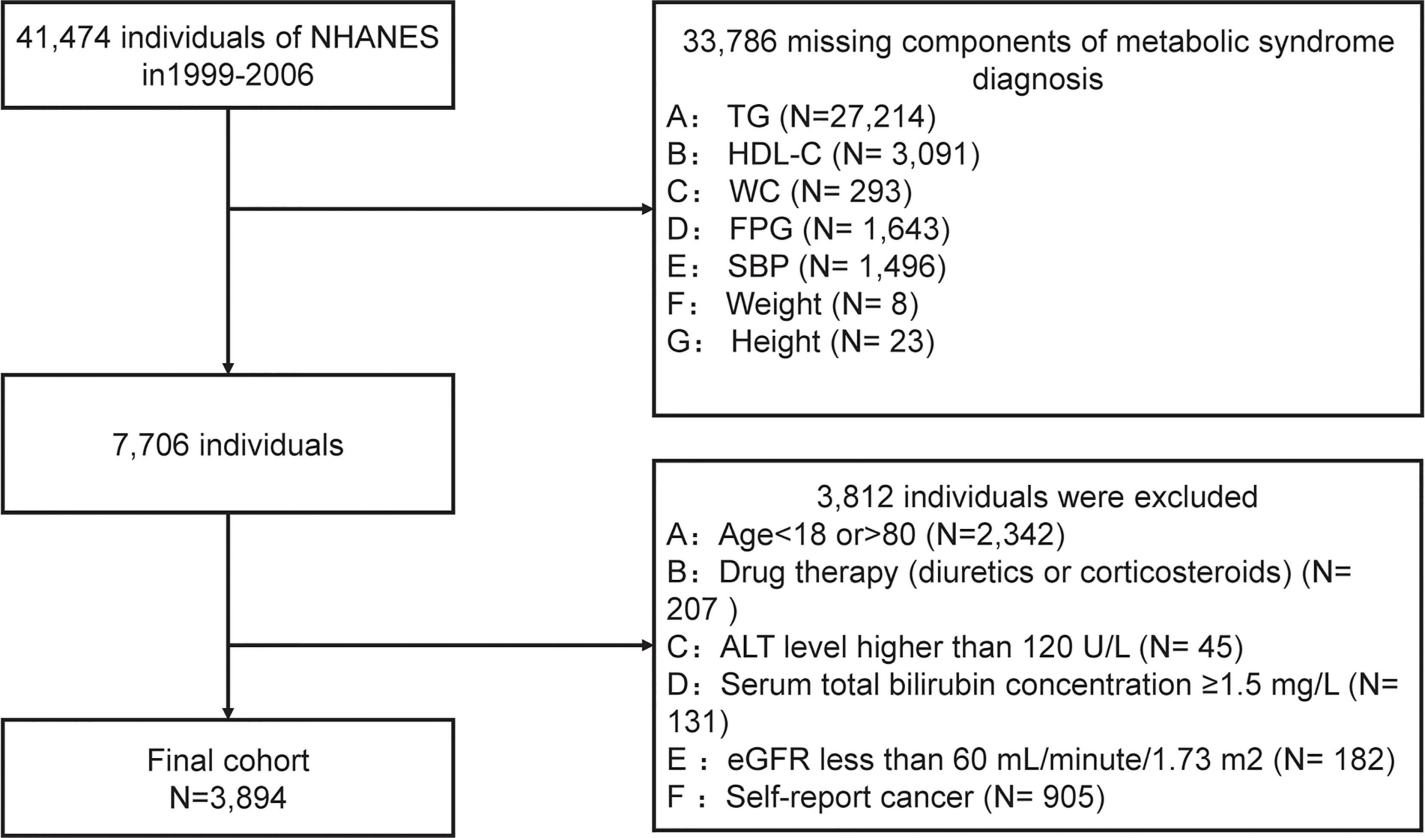
Flow chart.

We conducted a secondary data analysis based on data extracted from NHANES cycles: 1999-2000, 2001-2002, 2003-2004, and 2004-2006. After a series of screenings, 3894 subjects were included in the final data analysis. Subjects were filtered based on the following exclusion criteria, and were shown in **Figure 1**:

(1) subjects without components of metabolic syndrome data (n=33786);
(2) people aged < 18 years or aged >80 years (n=2342);
(3) drug therapy (diuretics or corticosteroids) that could influence weight (n=207);
(4) with a suspected renal or liver insufficiency: an estimated glomerular filtration rate (eGFR) ≤60 mL/minute/1.73 m^2^ (n=182) or serum total bilirubin concentration ≥1.5 mg/L (n=131), or alanine aminotransferase (ALT) level ≥120 U/L (n=45).
(5) any cancer or malignancy (n= 905).

### Anthropometric Indexes Measurement

Each participant had a home interview and finished a physical examination at a mobile examination center (MEC). Participants were required to fast at least 9 hours before the health examination (22). Height and weight were measured at the MEC by a standardized protocol. BMI was calculated by dividing the weight in kilograms by the square of the height in meters and then rounding to the nearest 1/10 cm. WC was measured by an inelastic ruler with a minimum scale of one millimeter at the end of a normal exhalation, and when standing naturally with legs opened about 25-30 cm apart. Placed the ruler at the midpoint of the connecting line between the upper edge of the top of the iliac crest and the lower edge of the 12th rib (usually the natural narrowest part of the waist) and horizontally circled the abdomen, and then rounded to 0.1cm (24). After at least 5 minutes of rest at the MEC, using a standardized mercury sphygmomanometer to measure blood pressure in a sitting position (25).

LAP, VAI, TyG, and WTI were calculated by using the following formulas (14, 20, 26, 27):


$$\begin{array}{l} \text{LAP }=\;\left[\text{WC }\left(\text{cm}\right)-65\right]\times \text{TG}\left(\text{mmol/L}\right)\text{ for male and }\left[\text{WC }\left(\text{cm}\right)-58\right]\times \text{ TG}\\ \left(\text{mmol/L}\right)\text{ for female} \end{array}$$

$$\begin{array}{l} \text{VAI }=\;\left[\text{WC }\left(\text{cm}\right)/39.68+\left(1.88\times \text{BMI}\left(\text{kg}/\text{m}2\right)\right)\right]\times \left(\text{TG}\left(\text{mmol/L}\right)/1.03\right)\\ \times \left(1.31/\text{HDL}-\text{C}\left(\text{mmol/L}\right)\right)\text{ for male and }[\text{ WC }\left(\text{cm}\right)/36.58+(1.89\times \text{BMI}\\ (\text{kg}/\text{m}2))]\;\left(\text{TG}\left(\text{mmol/L}\right)/0.81\right)\times \left(1.52/\text{HDL}-\text{C}\left(\text{mmol/L}\right)\right)\text{ for female} \end{array}$$

TyG = Ln [TG(mg/dL)× FPG (mg/dL)/2]

WTI = Ln [TG(mg/dL)×WC(cm)/2]

### Biochemical Measurements

Total cholesterol (TC), TG, low-density lipoprotein cholesterol (LDL-C), and HDL-C were estimated in subjects who fasted for at least 8.5 hours but less than 24 hours. Venous blood samples of participants were collected and processed in MECs following the NHANES protocols.

According to the established protocols, the samples were packed in cold bags or dry ice, and directly transported to the Collaborative Studies Clinical Laboratory by Federal Express and stored at-70°C for analysis (28). Johns Hopkins University School of Medicine Lipoprotein Analytical Laboratory tested the blood samples of lipid. Interlaboratory quality control carried out by the laboratories met the Centers for Disease Control and Prevention (CDC) program’s acceptable performance of allowable bias and imprecision.

HDL-C was determined using a nephelometric immunoassay on the Hitachi 717 Analyzer (Hitachi Global Storage Technologies, California). The FPG was measured using the enzyme hexokinase (HK) method. TG was measured using an automatic direct chemiluminescence analyzer (Beckman Synchron LX20, USA). All laboratory measurements met the requirements of the standardization and certification program. More detailed information about the analyzers and methods used can be obtained from the laboratory method file available on the NHANES website.

### MetS Definition

MetS was defined according to the updated National Cholesterol Education Program/Adult Treatment Panel III criteria (NCEP-ATP III) for Americans, that was, meeting the following three or more components: WC ≥102cm for male or ≥88cm for female; blood pressure ≥130/85mmHg or treated with anti-hypertensive drugs; or FPG ≥5.6 mmol/L or drugs used for treating diabetes; TG ≥150mg/dL or treated with drugs for this lipid abnormality; HDL-C <40mg/dL for male or <50mg/dL for female or treated with drugs for this lipid abnormality (29).

### Variables

In this study, the independent variables were VAI, LAP, TyG, and WTI respectively. The dependent variable was MetS. Covariates were prioritized according to the previous research on risk factors for MetS (29–32). Socio-demographic characteristics such as sex, education, race/ethnicity were collected. A self-reported questionnaire was applied to evaluate medication use (glucose-lowering drugs, lipid-lowering drugs, and anti-hypertensive drugs). According to the self-completed questionnaire, physical activities were divided into four categories (moderate, low, moderate, and high), and smoking was separated into current smokers, former smokers, and non-smokers. We also collected a series of laboratory data such as homocysteine, glucose, insulin, hs-CRP, TG, TC, HDL-C, LDL-C, albumin, total bilirubin, total protein, uric acid, and BUN, and a set of the dietary condition like alcohol intake, energy, total saturated fatty acids (TSFA), total polyunsaturated fatty acids (TPFA), total monounsaturated fatty acids (TMFA), total fat, protein. The physical activity categories were based on the distribution of MET-minute levels for the present NHANES sample. Diabetes was defined as a self-reported physician diagnosis of diabetes or a fasting glucose concentration >126 mg/dL. Hypertension was defined by ≥1 of the following criteria: systolic blood pressure ≥140 mmHg or diastolic blood pressure ≥90 mmHg or self-reported physician diagnosis of hypertension. Drink consumption is defined as 5 gm or more drinks per day. Insulin resistance (HOMA-IR) was calculated as following formula (33):


$$\begin{array}{l} \text{HOMA}-\text{ IR }=\;[\text{fasting insulin concentration }\left(\text{μIU/mL}\right)\times \text{FPG}\\ \left(\text{mml/L}\right)/22.5]\; \end{array}$$

### Statistical Methods

The statistical analysis was conducted by the guidelines of the CDC (https://www.cdc.gov/nchs/nhanes/tutorials/default.aspx). All analyses used EmpowerStats (http://www.empower.stats.com, X&Y Solutions, Inc., Boston, MA) and the statistical software packages R (http://www.R-project.org, The R Foundation R.3.4.3).

In this study, sample weights were adjusted according to the recommendations of the NCHS. To present nationally representative estimates, survey analysis procedures were used to account for the sample weights (MEC exam weight), stratification, and clustering of the complex sampling design (34). We calculated the sample weight for the 8 years of data from 1999 to 2006 as WT_99–06_ =  (1/4) × WT_05–06_+ (1/4) × WT_03–04_+(1/2) × WT_99–02_, WT_99–02_ is the variable WTMEC4YR from the NHANES 1999–2000 and NHANES 2001–2002; WT_03–04_and WT_05–06_ were the variable WTMEC2YR from the NHANES 2003–2004 and NHANES 2005–2006 demographic file, respectively (35, 36). Data were expressed as weighted proportions (± Standard Error (SE)) for categorical variables and as weighted means ± SE for continuous variables depending on their type. In estimating standard errors, the complex sample design was incorporated by using Taylor series linearization with provided survey design variables (37). We tested differences in characteristics between the MetS group and the non-MetS group with a one-way analysis of variance for continuous variables and with chi-square tests for categorical variables. Weighted logistic regression was applied to analyze the relationship between anthropometric indicators (VAI, LAP, TyG, WTI) and MetS. We selected these confounders on the basis of their associations with the MetS or a change in effect estimate of more than 10% (38). Further, the receiver operating characteristic (ROC) curve was drawn and the area under curve (AUC) value was calculated to evaluate the predictive ability of LAP, TyG, VAI, and WTI for MetS. DeLong et al’s non-parametric method was performed to compare the AUC between LAP and other indexes (32). According to the maximum value of the sum of sensitivity and specificity, the best cutoff values of LAP, VAI, TyG, and WTI for predicting MetS were determined. All statistical significance was set to *P*<0.05.

## Results

### Baseline Characteristics of the Subjects

As shown in **Figure 1**, according to the exclusion criteria, 3794 subjects (1893 males and 2001 females) were finally included in this study. **Table 1** describes the baseline characteristics of the population. At baseline, 560 (35.2%) males and 529 (26.4%) females were diagnosed with MetS. The average age of included subjects was 38.17± 0.47 years for the non-MetS group and 47.25 ± 0.42 years for the MetS group. Significantly higher levels of mean systolic, mean diastolic, glucose-plasma, TC, HOMA-IR, TG, LDL-C, uric acid, alanine aminotransferase, aspartate aminotransferase, and gamma-glutamyl transferase were observed in subjects with MetS. But they had significantly lower levels of HDL-C, albumin than those without MetS.

**Table 1:** Baseline characteristics of the participants.

	Non-MetS Group	MetS Group	*P* value
**Age** (yr)	38.17 ± 0.47	47.25 ± 0.42	<0.0001
**Sex**, %			0.0037
Female	50.86 ± 0.80	45.58 ± 1.85	
Male	49.14 ± 0.80	54.42 ± 1.85	
**Race**, %			0.006
Non-Hispanic Black	11.24 ± 1.10	7.28 ± 1.12	
Mexican American	8.82 ± 1.04	7.99 ± 1.15	
Other Hispanic	6.03 ± 1.22	6.47 ± 1.72	
Non-Hispanic White	69.1 ± 0.83	73.06 ± 2.62	
Other race	4.8 ± 0.62	5.2 ± 1.13	
**Education**, %			<0.0001
< high school	18.22 ± 1.13	21.56 ± 1.30	
High school	24.13 ± 1.20	29.91 ± 1.77	
> high school	57.65 ± 1.68	48.53 ± 1.74	
**Poverty to income ratio**	3.0 3 ± 0.07	3.11 ± 0.07	0.3338
**Smoking**, %			<0.0001
Never	51.84 ± 1.49	46.54 ± (2.17	
Former	20.42 ± 1.22	27.39 ± 1.68	
Current	27.74 ± 1.20	26.07 ± 1.65	
**Drink consumption**, %			<0.0001
No	70.35 ± 1.52	78.59 ± 1.51	
Yes	29.65 ± 1.52	21.4 1 ± 1.51	
**Physical activity**, %			0.013
Sedentary	15.9 ± 1.00	18.21 ± 1.71	
Low	28.22 ± 1.61	31.49 ± 2.12	
Moderate	20.63 ± 0.86	16.84 ± 1.71	
High	35.24 ± 1.38	33.46 ± 2.20	
**Medication use**			
**Glucose-lowering drugs**, %			<0.0001
No	99.43 ± 0.15	92.95 ± 0.92	
Yes	0.57 ± 0.15	7.05 ± 0.92	
**Lipid-lowering drugs**, %			<0.0001
No	98.19 ± 0.29	85.53 ± 1.17	
Yes	1.81 ± 0.29	14.47 ± 1.17	
**Antihypertensive drugs**, %			<0.0001
No	96.94 ± 0.43	79.58 ± 1.32	
Yes	3.06 ± 0.43	20.42 ± 1.32	
**Laboratory data**			
Triglyceride (mg/dL)	109.92 ±1.56	235.24 ± 9.63	<0.0001
LDL-cholesterol (mg/dL)	118.33 ±0.92	127.25 ± 1.73	<0.0001
Albumin (g/dL)	4.36 ±0.01	4.26 ± 0.01	<0.0001
Alanine aminotransferase ALT (U/L)	23.44 ±0.28	29.73 ± 0.7	<0.0001
Aspartate aminotransferase AST (U/L)	23.19 ±0.20	24.82 ± 0.49	0.003
Gamma glutamyl transferase (U/L)	24.37 ±0.53	37.2 ± 2.07	<0.0001
Glucose, serum (mg/dL)	89.43 ±0.40	106.81 ± 1.07	<0.0001
Total bilirubin (mg/dL)	0.69 ±0.01	0.68 ± 0.01	0.3674
Total protein (g/dL)	7.330 ± 0.02	7.29 ± 0.02	0.2184
Triglycerides (mg/dL)	101.23 ±1.64	222.2 ± 10.33	<0.0001
Uric acid (mg/dL)	5.05 ±0.02	5.83 ± 0.06	<0.0001
Plasma glucose (mmol/L)	5.19 ±0.02	6.23 ± 0.07	<0.0001
HDL-cholesterol (mg/dL)	54.37 ± 0.34	42.03 ± 0.60	<0.0001
HOMA-IR	2.09 ±0.04	5.12 ± 0.20	<0.0001
**Dietary**			
Energy (kcal)	2330.21±22.24	2255.53 ± 31.96	0.0984
Total monounsaturated fatty acids (gm)	32.54 ± 0.46	32.71 ± 0.69	0.8449
Total polyunsaturated fatty acids (gm)	18.01 ± 0.25	17.57 ± 0.41	0.3831
Protein (gm)	85.99 ± 0.96	85.05 ± 1.64	0.645
Total saturated fatty acids (gm)	28.43 ± 0.40	28.54 ± 0.62	0.8887
Total fat (gm)	86.78 ± 1.12	86.78 ± 1.60	0.9993
**Anthropometry**			
Weight (kg)	75.37 ± 0.38	92.64 ± 0.88	<0.0001
Standing height (cm)	169.79 ±0.24	170.84 ± 0.38	0.0113
Body mass index (kg/m^2)	26.07 ± 0.13	31.61 ± 0.25	<0.0001
Waist circumference (cm)	90.18 ± 0.32	106.83 ± 0.56	<0.0001
mean systolic	114.44 ±0.34	123.8 ± 0.55	<0.0001
mean diastolic	70.26 ± 0.27	75.2 ± 0.49	<0.0001
VAI	1.44 ± 0.03	4.33 ± 0.25	<0.0001
LAP	34.15 ± 0.77	110.23 ± 4.55	<0.0001
TyG.	8.32 ± 0.01	9.18 ± 0.03	<0.0001
WTI	8.29 ± 0.01	9.16 ± 0.03	<0.0001
**MetS Components**			
Elevated BP, %			<0.0001
No	86.86 ± 0.75	46.68 ± 1.81	
Yes	13.14 ± 0.75	53.32 ± 1.81	
Elevated TG level, %			<0.0001
No	88.26 ± 0.72	34.05 ± 1.84	
Yes	11.74 ± 0.72	65.95 ± 1.84	
Reduced HDL-C level, %			<0.0001
No	76.76 ± 1.05	21.47 ± 1.82	
Yes	23.24 ± 1.05	78.53 ± 1.82	
Drugs used for low level of high-density lipoprotein cholesterol, %			<0.0001
No	98.19 ± 0.29	85.76 ± 1.19	
Yes	1.81 ± 0.289	14.24 ± 1.19	
Drugs used for high level of triglyceride, %			0.0047
No	100 ± 0	99.72 ± 0.14	
Yes	0 ± 0	0.28 ± 0.14	
Elevated WC, %			
No	70.23 ± 1.18	15.16 ± 1.54	
Yes	29.77 ± 1.18	84.84 ± 1.54	
HDM, %			<0.0001
No	85.41 ± 0.89	32.8 ± 2.12	
Yes	14.59 ± 0.89	67.2 ± 2.12	

Data are expressed as weighted proportions [± Standard Error (SE)] for categorical variables and as weighted means ± Standard Error for continuous variables depending on its type. Variables between groups with and without MetS were compared using one-way analysis of variance for continuous variables and with chi-square tests for categorical variables.

In addition, as shown in **Table 1**, all of the anthropometric indexes in the MetS group, including VAI, LAP, TyG, WTI, BMI, WC, height, BMI, and weight were significantly increased.

### The Anthropometric Indexes for Predicting MetS

**Figure 2:**
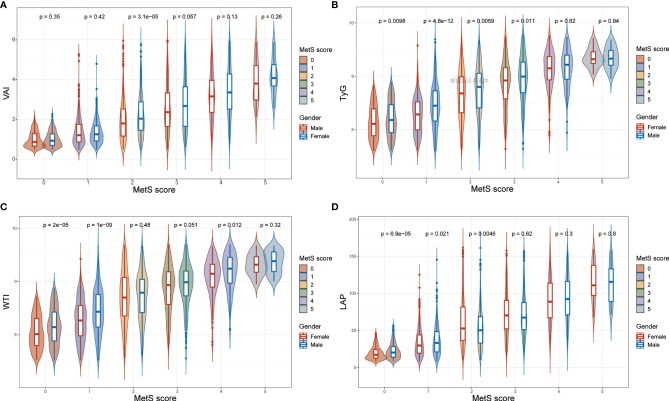
The values of **(A)** visceral adiposity index (VAI), **(B)** triglycerides and glucose (TyG), **(C)** waist-triglyceride index and **(D)** lipid accumulation product (LAP) according to MetS components in both genders.

**Figure 3:**
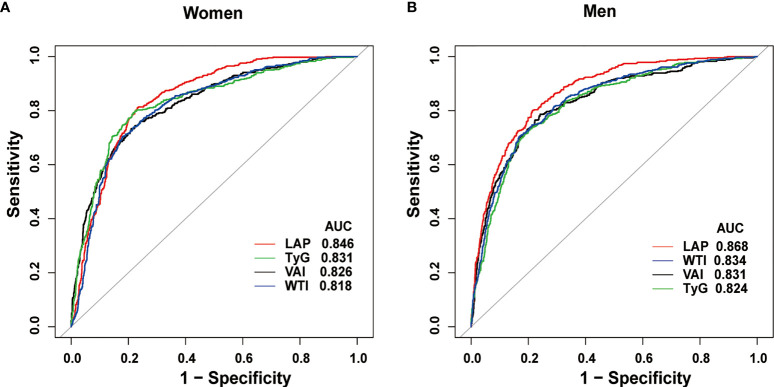
Receiver operating characteristic curves of LAP and other indexes in women **(A)** and men **(B)** for identifying MetS.

What can be seen in **Figure 2** is LAP, VAI, TyG, and WTI increased in proportion to the number of MetS components. **Table 2** and **Figure 3** show the AUC values [95% confidence interval (CI)] of the anthropometric indexes used to screen American adults with MetS. Of the four indexes examined, the highest AUC was LAP, 0.8458 for women (95% CI: 0.8272-0.8645) and 0.8685 for men (95% CI: 0.8504-0.8865). The optimum cutoff values of LAP predicted were 52.4291 (sensitivity 0.8117, specificity 0.7677) in women and 53.3125 (sensitivity 0.8013, specificity 0.7852) in men. The optimal cut-off points for TyG were 8.8221 in men and 8.6897 in women. This study also reported other details of all the anthropometric indexes such as negative predictive value (NPV) and positive predictive value (PPV) in **Table 2**.

**Table 2:** The anthropometric indexes for predicting Mets.

	Test	AUC	95%CI low	95%CI upp	Cutoff Value	Specificity	Sensitivity	PPV	NPV
Women	VAI	0.8261	0.8038	0.8483	2.0786	0.7901	0.7332	0.5274	0.9026
	LAP	0.8458	0.8272	0.8645	52.4291	0.7677	0.8117	0.5403	0.9238
	TyG	0.8315	0.8093	0.8537	8.6897	0.7770	0.7983	0.5527	0.9178
	WTI	0.8179	0.7961	0.8396	8.8231	0.8206	0.7043	0.5787	0.8880
Men	VAI	0.8309	0.8088	0.8530	1.8196	0.7590	0.7862	0.5656	0.8989
	LAP	0.8685	0.8504	0.8865	53.3125	0.7852	0.8013	0.5940	0.9097
	TyG	0.8237	0.8016	0.8458	8.8221	0.7937	0.7330	0.5668	0.8898
	WTI	0.8335	0.8121	0.8550	8.8820	0.8296	0.7063	0.6078	0.8831
Overall	VAI	0.8263	0.8106	0.8420	2.0798	0.8063	0.7183	0.5691	0.8893
	LAP	0.8565	0.8435	0.8695	53.3255	0.7805	0.7989	0.5689	0.9146
	TyG	0.8279	0.8123	0.8435	8.8478	0.8337	0.7096	0.5718	0.8901
	WTI	0.8251	0.8098	0.8404	8.8233	0.8036	0.7256	0.5718	0.8901

### Comparison of AUC Values Between LAP and Other Indexes in Men and Women

**Table 3** shows the differences in AUC values between LAP and other indexes for screening MetS. In men, differences in AUC values between LAP and TyG, WTI, VAI were significant (all *P*<0.001). In women, the AUC value between LAP and WTI was significantly different (*P*<0.001), but the statistical difference between LAP and TyG, VAI was not significant. The above results showed that LAP had a stronger ability to identify MetS than other anthropometric indexes.

**Table 3:** Comparison of AUC values between LAP and other indexes in both genders.

	Difference between Area (95%CI)	*P*-value
Women		
LAP *vs* TyG	0.0016 (-0.0015-0.0046)	0.8409
LAP *vs* WTI	0.0215 (0.0187-0.0243)	<0.001
LAP *vs* VAI	0.0087 (0.0055-0.0120)	0.2628
Men		
LAP *vs* TyG	0.0553 (0.0506-0.0600)	<0.001
LAP *vs* WTI	0.0429 (0.0391-0.0468)	<0.001
LAP *vs* VAI	0.0521 (0.0469-0.0573)	<0.001

Delong. Clarke-Pearson’s nonparametric approach was used to compare the AUCs of indexes.

### Associations Between Four Anthropometric Indexes and MetS

**Table 4** shows the adjusted odds ratios (ORs) (95% CIs) of anthropometric indexes for MetS in women and men. After adjusting for age, education, alcohol, current or a past cigarette smoker, poverty to income ratio, physical activity, uric acid, energy intake, protein intake, TMFA intake, TPFA intake, TSFA intake, total fat intake, glucose-lowering drugs, lipid-lowering drugs and anti-hypertensive drugs, the prevalence of MetS is higher in the third and fourth quartiles (Q3 and Q4) of LAP, TyG, VAI, and WTI in women. For VAI, Q3 was at 6.084 (2.320, 15.955) and Q4 was at 71.681 (26.334, 195.112), showing a higher risk for MetS compared to Q1. Q3 of LAP was at 24.174 (5.690, 102.698) and Q4 was at 199.843 (46.394, 860.825), which indicated that MetS was risker than the first quartile (Q1) of LAP. For TyG, Q3 was at 6.058 (2.871, 12.783) and Q4 was at 37.708 (17.214, 82.598), revealing that the risk of MetS was higher than Q1. For WTI, Q3 was at 4.747 (2.269, 9.929) and Q4 was at 38.472 (17.723, 83.513), indicating a higher risk for MetS compared to Q1.

**Table 4:** Associations between MetS and LAP, VAI, TyG and WTI.

Exposure	Adjusted Odds Ratio (95%CI)	*P*-value	Adjusted Odds Ratio (95%CI)	*P*-value
	Women		Men	
VAI (continuous variable)	4.174 (3.224, 5.404)	<0.001	3.375 (2.716, 4.194)	<0.001
VAI Quartile				
Q1 0.479-0.968	Reference		Reference	
Q2 0.968-1.543	2.838 (1.044, 7.716)	0.041	2.288 (0.969, 5.407)	0.059
Q3 1.544-2.510	6.084 (2.320, 15.955)	<0.001	5.622 (2.532, 12.482)	<0.001
Q4 2.510-5.963	71.681 (26.334, 195.112)	<0.001	36.702 (16.414, 82.067)	<0.001
*P* for trend	<0.001		<0.001	
LAP (continuous variable)	1.047 (1.038, 1.056)	<0.001	1.052 (1.043, 1.061)	<0.001
LAP Quartile				
Q1 8.337-22.744	Reference		Reference	
Q2 22.761-40.577	11.817 (2.734, 51.083)	<0.001	4.865 (1.245, 19.005)	0.023
Q3 40.626-68.526	24.174 (5.690, 102.698)	<0.001	23.137 (6.320, 84.704)	<0.001
Q4 68.542-161.564	199.843 (46.394, 860.825)	<0.001	125.125 (33.737, 464.075)	<0.001
*P* for trend	<0.001		<0.001	
TyG (continuous variable)	27.128 (14.724, 49.983)	<0.001	14.796 (8.771, 24.961)	<0.001
TyG Quartile				
Q1 7.593-8.163	Reference		Reference	
Q2 8.164-8.545	1.582 (0.712, 3.513)	0.260	3.452 (1.266, 9.407)	0.015
Q3 8.546-8.952	6.058 (2.871, 12.783)	<0.001	6.354 (2.476, 16.306)	<0.001
Q4 8.954-9.785	37.708 (17.214, 82.598)	<0.001	38.935 (15.069, 100.601)	<0.001
*P* for trend	<0.001		<0.001	
WTI (continuous variable)	20.556 (11.610, 36.395)	<0.001	20.115 (11.454, 35.325)	<0.001
WTI Quartile				
Q1 7.514-8.132	Reference		Reference	
Q2 8.133-8.543	1.535 (0.697, 3.378)	0.287	2.109 (0.759, 5.861)	0.153
Q3 8.543-8.968	4.747 (2.269, 9.929)	<0.001	5.976 (2.346, 15.228)	<0.001
Q4 8.969-9.698	38.472 (17.723, 83.513)	<0.001	38.645 (15.085, 99.003)	<0.001
*P* for trend	<0.001		<0.001	

Adjusted for age (years); race; education; alcohol; smoker; poverty to income ratio; physical activity; uric acid; energy; total monounsaturated fatty acids; total polyunsaturated fatty acids; protein; total saturated fatty acids; total fat; glucose-lowering drugs; lipid-lowering drugs; antihypertensive drugs.

In the fully adjusted model in men, each 1 unit increase in VAI increased the MetS risk by 237.5%. LAP increased by 1 unit, the incidence of MetS increased by 5.2%. The fully adjusted OR (95%CI) for TyG and WTI in men, respectively, were 14.796 (8.771, 24.961) and 20.115 (11.454, 35.325).

For sensitivity analysis, we converted VAI, LAP, TyG, WTI from continuous variables to categorical variables. The *P* for the trend of VAI, LAP, TyG, WTI with categorical variables was consistent with the result when VAI, LAP, TyG, WTI was a continuous variable.

## Discussion

This study assessed the capability of four low-cost, non-invasive and easily-calculated anthropometric indicators, including VAI, LAP, TyG, and WTI, to predict MetS. In this cross-sectional analysis of American adults, LAP, VAI, TyG, and WTI were significantly associated with MetS in both genders. Furthermore, ROC curve analysis showed that all parameters could distinguish subjects with MetS, and the AUC values were higher than 0.7 in both genders, of which LAP showed the greatest diagnostic accuracy.

To the best of our knowledge, this was the first study to explore the relationship between anthropometric parameters (LAP, VAI, TyG, and WTI) and MetS in the American population according to different genders, and their ability to diagnose MetS has been further evaluated.

LAP is reported to be associated with MetS, cardiovascular disease, and type 2 diabetes (13, 39, 40). Among these four indicators, LAP has the advantages of simplicity, low cost, and wide applicability to different populations. Shin et al. (17). reported that among the middle-aged and elderly people (aged 40 years or older) in South Korea, LAP was the best index for predicting MetS comparing with VAI, WHtR, and TyG. Similar results were observed in subsequent studies, which compared more different anthropometric indicators (41). In the present study, the best threshold of LAP for predicting MetS was 53.3125 in males and 52.4291 in females. In Argentinian healthy individuals, a similar value was achieved (53.63 in all subjects). However, a slightly lower best threshold was suggested in the Iranian population (49.71 for females and 39.89 females) (42) and in Spanish adults (48.09 for males and 31.77 for females) (43).The optimal threshold of MetS predicted by LAP is different from other studies, which may be due to the ethnic modification of insulin resistance and abdominal fat distribution, age of the enrolled population, as well as the diagnostic criteria of MetS used.

VAI is an important indicator for insulin resistance and visceral obesity and is associated with CVD risk (14, 19). In this study, the AUC values of VAI predicting MetS were 0.8309 and 0.8261 in males and females, respectively. The best cutoff point for VAI to predict MetS in female was 2.0786 and in male was 1.8196, close to the optimal critical point 2 of VAI for predicting MetS in the middle-aged and elderly in China (44). However, the cutoff in overweight and obese Turkish patients was higher with a value of 2.205 (45).

The results of our analysis also revealed the relatively high usefulness of TyG in identifying individuals with MetS. TyG is an index that combines FPG and TG and is considered to be a substitute for insulin resistance. Its ability to identify MetS has been confirmed by several studies. In middle-aged and elderly Chinese (44), TyG was suggested to be a credible surrogate marker for identifying MetS with the AUC of 0.802, and the best cut-off values were 8.9 and 8.7 for males and females, respectively. In the present population, the optimal cut-off values for males and females were 8.8221 and 8.6897 respectively, which was similar to their results. Furthermore, in the present study, the difference between TyG and LAP in predicting MetS of women was not significant.

In both genders, the predictive ability of WTI was significantly different from that of LAP, which suggested that WTI was weaker than LAP in predicting MetS. Inspired by the TyG, WTI was first proposed by Liu et al. (20). Their study showed that WTI and MetS risk in the Chinese population was associated, the AUC of WTI predicting MetS is 0.881 in women and 0.830 in men. In the present study, with the increase of the number of components of MetS, the value of WTI increases gradually. WTI has a good ability to predict MetS, and AUC is 0.8335 and 0.8179 in men and women, respectively, although it is weaker than LAP. And considering that the formulas of WTI and LAP are both combinations of TG and WC, and the calculation of WTI is more complex, WTI may not be the best index in identifying MetS.

### Strength and Limitations

One advantage of the study is that, first and foremost, the analysis included several confounders associated with MetS, such as smoking, alcohol consumption, physical activity, dietary intake, and socioeconomic factors. Moreover, the data analyzed in this study were from the NHANES database, which was national and representative in scope, the anthropometric data and laboratory data are of high quality.

The limitations of this study need to be pointed out (1). This was a cross-sectional study and cannot draw any conclusions about the anthropometric index changes over time (2). The study was limited to American adults, the applicability of these results to other populations may be limited. (3) For females, due to the lack of data on menopause, their menopausal status cannot be taken into account in data analysis. (4) This study defined MetS using NCEP-ATP III criteria. Thus, whether a consistent conclusion can be obtained under other criteria requires further studies. (5) A common problem in observational studies is unmeasured confunders. Although we have adjusted many potential confounding factors, we can’t rule out the possibility of residual confounding caused by unmeasured or unidentified factors.

The results of this study showed that LAP, VAI, TyG, WTI were reliable predictors of MetS for American adults, and LAP has the largest AUC in predicting MetS. Among females, the difference in AUC between LAP and TyG, VAI was not significant. We suggest that LAP is a useful screening indicator to identify MetS at a minimum cost in the clinical setting, considering the superiority and simplicity of LAP in identifying MetS.

## Conclusions

The present study indicated that LAP is a better predictor in the clinical setting for identifying individuals with MetS in the US adult population.

## Data Availability Statement

The original contributions presented in the study are included in the article/supplementary material. Further inquiries can be directed to the corresponding author.

## Ethics Statement

The studies involving human participants were reviewed and approved by The National Center for Health Statistics (NCHS) Ethics Review Board. The patients/participants provided their written informed consent to participate in this study.

## Author Contributions

Conceptualization: YL and TS; Methodology: RZ; Software: RZ; Validation: SL, RC, and HZ; Formal analysis: RZ, FN, and TS; Writing—original draft preparation: YL and RH; Writing—review and editing: YL and TS; All authors contributed to the article and approved the submitted version.

## Conflict of Interest

The authors declare that the research was conducted in the absence of any commercial or financial relationships that could be construed as a potential conflict of interest.

## Publisher’s Note

All claims expressed in this article are solely those of the authors and do not necessarily represent those of their affiliated organizations, or those of the publisher, the editors and the reviewers. Any product that may be evaluated in this article, or claim that may be made by its manufacturer, is not guaranteed or endorsed by the publisher.

## Abbreviations

BMI, body mass index; SBP, systolic blood pressure; DBP, diastolic blood pressure; TMFA, total monounsaturated fatty acids; TPFA, total polyunsaturated fatty acids; TSFA, total saturated fatty acids; eGFR, estimated glomerular filtration rate; HDL-C, high density lipoprotein cholesterol; CRP, C-reactive protein; LAP, lipid accumulation product; VAI, visceral adiposity index; TyG, triglyceride and glucose index; WTI, waist-triglyceride index; AUC, area under the curve.
